# 2.4 GHz CMOS Power Amplifier with Mode-Locking Structure to Enhance Gain

**DOI:** 10.1155/2014/967181

**Published:** 2014-06-17

**Authors:** Changhyun Lee, Changkun Park

**Affiliations:** School of Electronic Engineering, College of Information Technology, Soongsil University, 551 Sangdo-Dong, Dongjak-Gu, Seoul 156-743, Republic of Korea

## Abstract

We propose a mode-locking method optimized for the cascode structure of an RF CMOS power amplifier. To maximize the advantage of the typical mode-locking method in the cascode structure, the input of the cross-coupled transistor is modified from that of a typical mode-locking structure. To prove the feasibility of the proposed structure, we designed a 2.4 GHz CMOS power amplifier with a 0.18 *μ*m RFCMOS process for polar transmitter applications. The measured power added efficiency is 34.9%, while the saturated output power is 23.32 dBm. The designed chip size is 1.4 × 0.6 mm^2^.

## 1. Introduction

Currently, CMOS (complementary metal-oxide semiconductor) devices are the most popular for RFIC (radio frequency integrated circuit) design due to their low cost [1–15]. In particular, CMOS RFICs can more easily be integrated with other analog or digital ICs than with GaAS (gallium arsenide) RFICs [16–21]. Although GaAs devices are regarded as more suitable than CMOS ones, there have been vigorous studies about how to reduce unit costs of CMOS power amplifiers [22–27]. If a successful CMOS power amplifier is developed, the potential for creating a fully integrated, front-end IC should increase. Nevertheless, compared to those using GaAs, CMOS power amplifiers still have drawbacks, including (1) low breakdown voltage, (2) lossy substrate, (3) low linearity, and (4) low gain. The issues related to the breakdown voltage and substrate loss have been successfully investigated and resolved using the distributed active transformer (DAT) proposed by Aoki et al. [22]. Additionally, techniques to solve the low-linearity problem of CMOS power amplifiers have also been intensively studied, and some successful techniques have been introduced [28–31].

Regarding the issue of low gain of CMOS power amplifiers, the mode-locking technique is one of the most successful solutions [32]. Accordingly, the concepts of the mode-locking technique have been vigorously adapted in previous work. In this study, we also focused on the improvement of gain of the CMOS power amplifier. While the mode-locking technique was adapted to a common-source amplifier in previous work, here, we propose a method for the mode-locking technique to be adapted to the cascode structure. The cascode structure is essential to overcome the low breakdown voltage problems of CMOS devices. To prove the feasibility of the proposed structure, we designed a 2.4 GHz CMOS power amplifier using the proposed structure.

## 2. Typical Mode-Locking Technique

**Figure 1:**
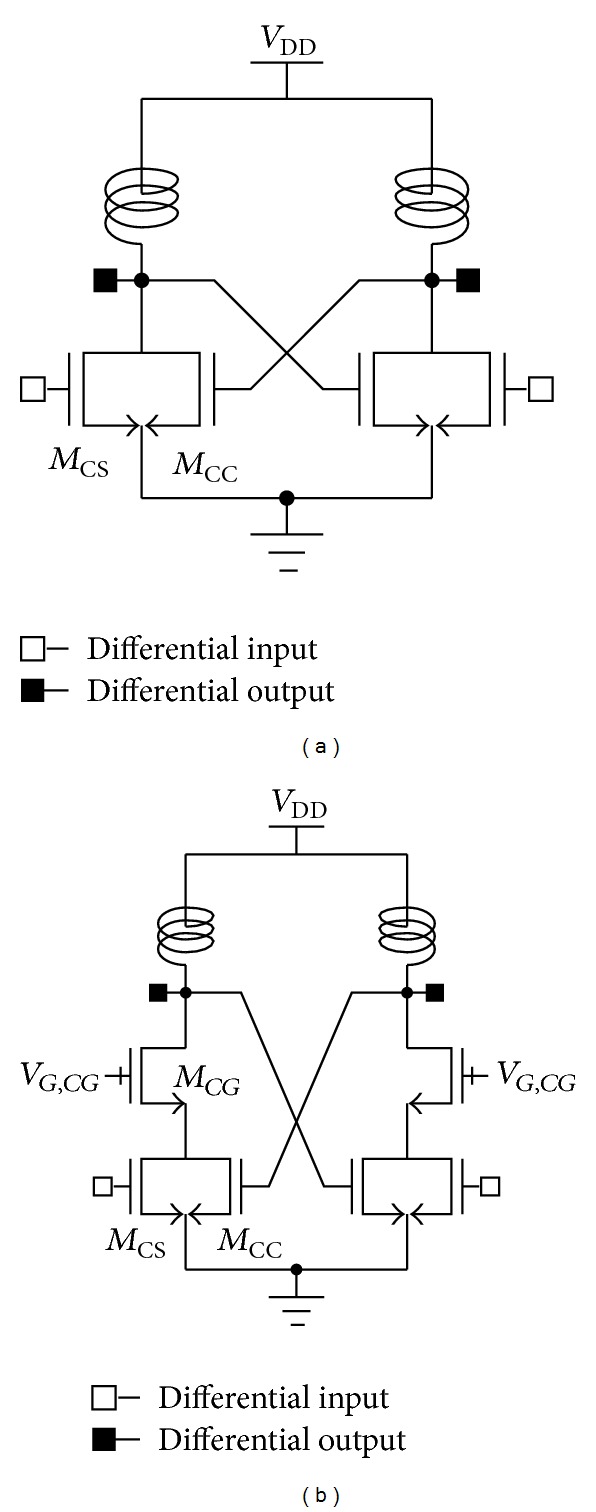
CMOS power amplifiers using mode-locking technique: (a) typical and (b) modified structures.

Figure 1 provides examples of CMOS power amplifiers using typical mode-locking technique. The structure shown in Figure 1(a) is the primary structure of the amplifier using the mode-locking technique. In Figure 1, for the sake of simplicity, the switch to control the oscillation is omitted. As shown in Figure 1(a), the differential structure is essential to adapt the mode-locking technique. Moreover, the differential structure provides an advantage for generating a virtual ground node and hence for minimizing the gain-reduction problems induced by the bond wires. As can be seen in Figure 1(a), the cross-coupled transistors (*M*_CC_) were used to construct the mode-locking structure. Although the input signal enters through the gate of the common-source transistors (*M*_CS_), the *M*_CC_ also acts as the amplifier stage. Accordingly, the mode-locking structure can elevate the gain as compared to a typical common-source amplifier.

Recently, as the CMOS technology has been scaled down, the cascode structure has become the most commonly used one for CMOS power amplifiers, to moderate breakdown voltage problems.  Figure 1(b) shows the cascode structure adapted for the mode-locking technique. In Figure 1, the drain voltage of *M*_*CG*_ is used as the input of *M*_CC_. In a previous work [33], to moderate the excessive voltage swing of input of *M*_CC_, the series capacitor was inserted between the drain of *M*_*CG*_ and the gate of *M*_CC_. However, the conceptual operation principle presented in Figure 1(b) is identical to that in Figure 1(a).

## 3. Proposed Mode-Locking Method with the Cascode Structure

**Figure 2:**
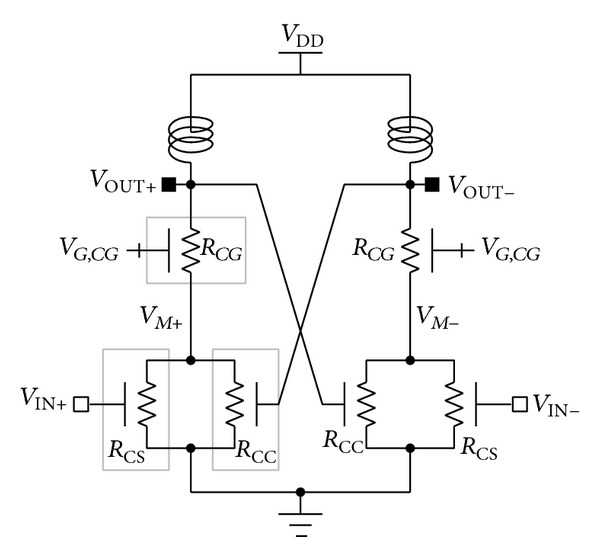
Simple equivalent circuit of cascode structure with mode-locking method.

Although the feasibility of the mode-locking technique merged into the cascode structure was successfully proven in previous work [33], the time delay between input of *M*_CS_ and input of *M*_CC_ of the structure shown in Figure 1(b) may obstruct maximization of the advantages of the mode-locking technique. To investigate the time delay problems indicated in Figure 1(b), we simplified the structure shown there with on-resistances as shown in Figure 2. In Figure 2, *R*_CS_, *R*_*CG*_, and *R*_CC_ denote the on-resistances of *M*_CS_, *M*_*CG*_, and *M*_CC_, respectively. If the time delay between *V*_IN+_ (or *V*_IN−_) and *V*_*M*+_ (or *V*_*M*−_) is *t*_CS_, the time delay, *t*_CC_, between *V*_IN+_ (or *V*_IN−_) and *V*_OUT+_ (or *V*_OUT−_) can then be calculated as follows:

(1) $$\begin{array}{c} t_{\text{CC}}\approx t_{\text{CS}}+5\tau \;\left(\tau =R_{CG}C_{\text{OUT}}\right). \end{array}$$

**Figure 3:**
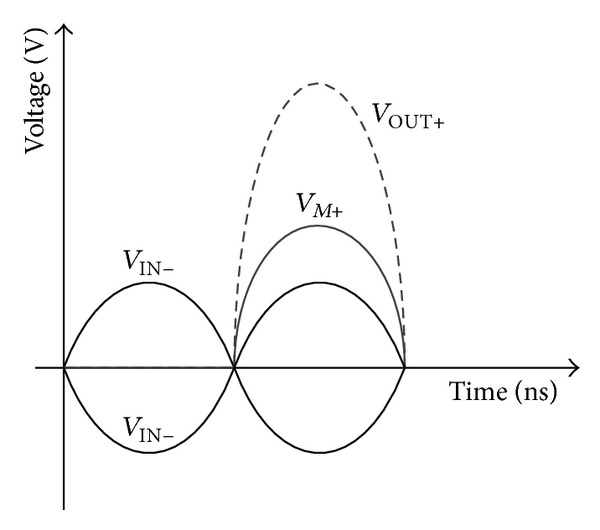
Ideal voltage waveforms of the cascode structure with mode-locking method.

Here, *C*_OUT_ is the equivalent capacitance at *V*_OUT+_ or *V*_OUT−_. In (1), we ignored effects induced by the load impedances connected to *V*_OUT+_ and *V*_OUT−_. If the effects of load impedances are considered, the time constant, *τ*, increases. Additionally, we assumed that the *C*_OUT_ is fully discharged or charged after five time constants.  Figure 3 provides the ideal voltage waveforms of the device in Figure 2.

Given that *M*_CC_ should perform the identical function of the *M*_CS_ in general, the value of *t*_CC_ needs to be minimized to maximize the advantage of the mode-locking technique. Undesired, excessive time delay, *t*_CC_, may cause the undesired effects, even harmonics. Additionally, the excessive value of *t*_CC_ may prevent switching conditions that would be ideal for high efficiency of the switching-mode power amplifier.

**Figure 4:**
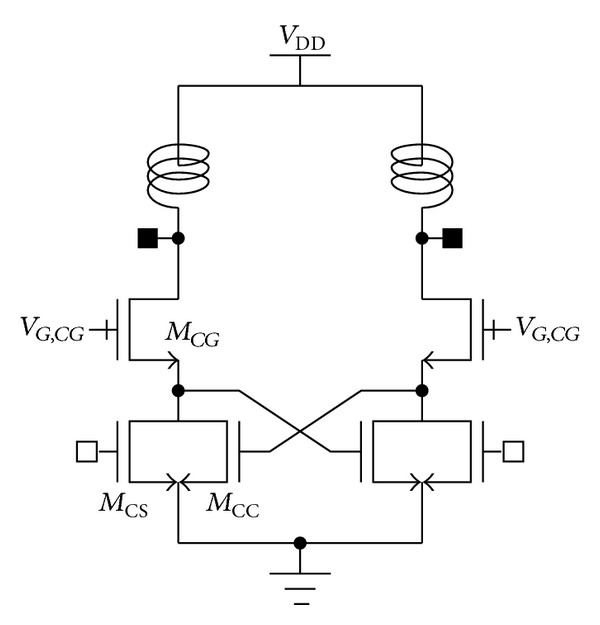
Proposed mode-locking technique for the cascode structure.

Here, we proposed a modified, mode-locking technique for the cascode structure to minimize the time delay, *t*_CC_ of (1). In the proposed structure (Figure 4), the input of the *M*_CC_ is connected to the drain of *M*_CS_. The time delay between input of *M*_CS_ and input of *M*_CC_ is reduced to *t*_CS_.

Compared to the typical structure shown in Figure 1(b), the time delay is reduced with amount of 5*τ* of (1). Although the time delay, *t*_CS_, still exists, the undesired effects induced by the excessive time delay may be minimized with the proposed structure.

## 4. Experimental Results: Design and Measured Results of 2.4 GHz CMOS Power Amplifier with Proposed Mode-Locking Technique

**Figure 5:**
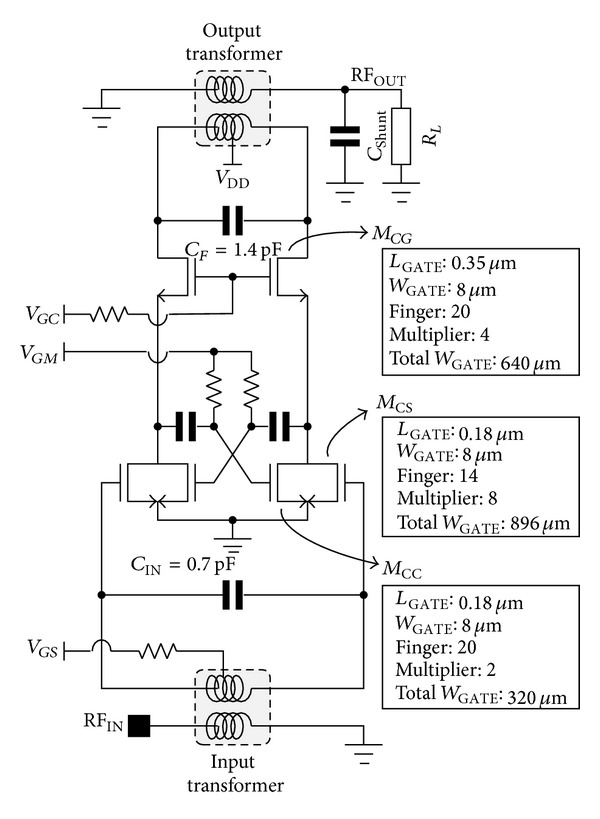
Schematic of the proposed power amplifier.

**Figure 6:**
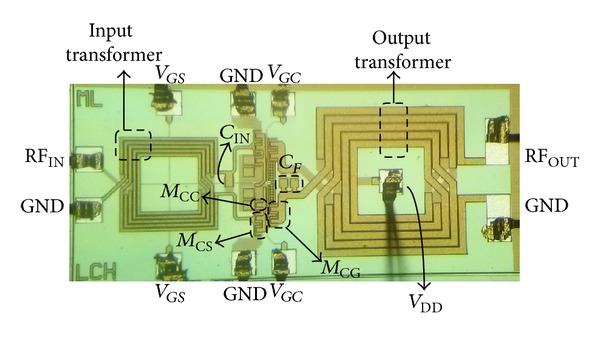
Photograph of the newly designed power amplifier.

To verify the feasibility of the proposed structure, we designed a 2.4 GHz power amplifier using 0.18 *μ*m RF CMOS technology with one poly, and six metal layers. Top metal layer was composed of aluminum 2.3 *μ*m thick. The power amplifier is designed as switching mode amplifier for polar transmitter, or sensor network, applications. All of the input and output matching networks are fully integrated, including test PADs and transformers. Important design parameters, including the transistor size, are provided in Figure 5. The input and output transformer were designed using an electromagnetic simulator. To minimize the loss induced by the resistance of the output transformer, the width of the output transformer is wider than that of the input transformer. The supply voltage of the amplifier enters through the center tap of the primary part of the output transformer. To minimize the gain reduction problems induced by the bond wires, a differential structure was adapted. All of the resistors for the bias are 2 kΩ.  Figure 6 shows the chip photograph of the newly designed power amplifier. The chip size is 1.4 × 0.6 mm^2^.

**Figure 7:**
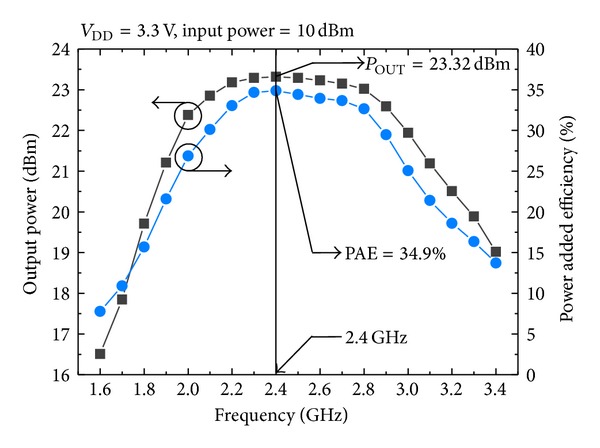
Measured output power and efficiency according to operating frequency.

**Figure 8:**
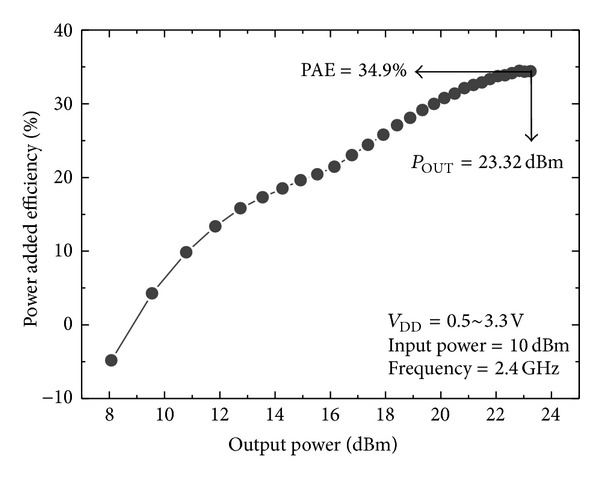
Measured output power and efficiency according to supply voltage.

Figure 7 shows the measured output power and power added efficiency (PAE), according to the operating frequency, with a fixed supply voltage (*V*_DD_) of 3.3 V. As provided in Figure 7, the output power and PAE at 2.4 GHz were 23.32 dBm and 34.9%, respectively.  Figure 8 shows the PAE versus the output power according to *V*_DD_ ranging from 0.5 V to 3.3 V.

## 5. Conclusions

In this study, we proposed a mode-locking technique for a cascode CMOS power amplifier. Using the drain voltage of a common-source transistor as the input of the cross-coupled transistor, the time delay between the common-source and cross-coupled transistors was minimized to maximize the advantage of the mode-locking technique. To prove the feasibility of the proposed technique, we designed a 2.4 GHz CMOS power amplifier with a 0.18 *μ*m RFCMOS process for polar transmitter applications. The measured power added efficiency is 34.9%, while the saturated output power is 23.32 dBm. The size of the newly designed chip was 1.4 × 0.6 mm^2^.
